# A Study of the Quantitative Relationship between Yield Strength and Crystal Size Distribution of Beeswax Oleogels

**DOI:** 10.3390/gels8010039

**Published:** 2022-01-05

**Authors:** Varuzhan Sarkisyan, Roman Sobolev, Yuliya Frolova, Irina Vorobiova, Alla Kochetkova

**Affiliations:** Laboratory of Food Biotechnology and Foods for Special Dietary Uses, Federal State Budgetary Scientific Institution Federal Research Centre of Nutrition, Biotechnology and Food Safety, 109240 Moscow, Russia; sobolevrv@bk.ru (R.S.); y.operarius@yandex.ru (Y.F.); vorobiova@ion.ru (I.V.); kochetkova@ion.ru (A.K.)

**Keywords:** oleogel, beeswax, molecular crystal, microstructure entropy, Hall-Petch relation, yield strength

## Abstract

Beeswax and beeswax hydrocarbon-based oleogels were studied to evaluate the quantitative relationship between their yield strength and crystal size distribution. With this aim, oleogels were prepared using four different cooling regimes to obtain different crystal size distributions. The microstructure was evaluated by polarized light microscopy. The yield strength is measured by the cone penetration test. Oleogels were characterized by average grain size, microstructure entropy, grain boundary energy per unit volume, and microstructure temperature. We have provided the theoretical basis for interpreting the microstructure and evaluating the microstructure-based hardening of oleogels. It is shown that the microstructure entropy might be used to predict the yield strength of oleogels by the Hall-Petch relationship.

## 1. Introduction

Edible oleogels are in a focus of researchers for the last two decades as a potential replacer of trans-isomeric and saturated fatty acids in foods. Oleogels are considered to be substantially diluted materials with rheological properties of solid with some interconnected structure that is responsible for these properties [1]. Ethylcellulose, hydroxystearic acid, sterols, paraffines, and waxes are usually used as gelling agents [2]. In the case of wax-based oleogels, the solid interconnected structure is formed by crystals of low-molecular-weight organic gelators (LMOG) from wax (hydrocarbons, wax esters, free fatty acids, and free fatty alcohols) [3,4]. The gelation mechanisms differ between these LMOGs. The main mechanisms driving the gelation by waxes are hydrogen bonding, van der Waals interactions, and π–π-stacking. Besides, LMOGs are usually self-organized and oriented according to the location of the hydrophobic and hydrophilic groups. On the contrary, in the case of hydrocarbon-based oleogels, only van der Waals forces lead to gelation [5].

Tailoring the rheological and textural properties of oleogels is one of the most promising research directions. Oleogels are usually described by hardness, yield strength, and also storage and loss modulus [6]. These parameters substantially depend on the technological regimes of processing. For example, it was shown that increasing the cooling rate positively affects the oleogel firmness. Similar changes are also observed for the oleogel microstructure, particularly in decreasing the crystal size [7,8,9,10]. Despite a considerable number of articles on the study of the structure and physical properties of oleogels, there is still no common physically substantiated understanding of the relationship between structure and properties of wax-based oleogels. One of the most thorough works in this direction was conducted by Blake et al. [11]. This work demonstrates the existence of statistical relationships between these parameters. Authors have shown that the small crystal size leads to their homogeneous distribution. They have also hypothesized that smaller pore sizes and their more uniform distribution increase the oil binding capacity due to higher total surface areas. This approach is shown to be effective, but it lacks in explaining the mechanism underlying the interaction between oleogels’ crystals. However, the theory on the relationship between structure and properties of polycrystalline materials (for example, metals) has been extensive developed for past decades [12]. The central idea of this theory is the empirically established (by Hall [13] and Petch [14]) relation between that the yield strength and the grain size, according to the Equation (1) (Hall-Petch equation)

(1) $$E_{s}=E_{0}+\frac{k}{\sqrt{\bar{d}}}$$

where $E_{s}$—yield strength (kPa), $\bar{d}$—average grain diameter of a polycrystalline material (mm), $E_{0}$—macro elasticity limit (kPa), and k—grain boundary hardening coefficient (kPa × mm^1/2^), characterizing the contribution of grain boundary to hardening.

The coefficients $E_{0}$ and k are assumed to be constant for each type of material in a range of grain sizes varying from 1 mm down to 1 μm [15]. For this range of grain sizes, this equation implies an increase in yield strength associated with a decrease in average grain diameter. According to Hall, grain boundaries are considered as obstacles preventing dislocation motion in polycrystalline material. Thus, the smaller the grain size, the larger the contact area of the grain boundaries, and therefore the material becomes harder.

Recently it was shown [16] that yield strength might be characterized not only by grain size but also by microstructure entropy ($S_{m}^{*}$) through the modified Hall-Petch Equation (2).

(2) $$E_{s}=E_{0}+\frac{k}{\sqrt{S_{m}^{*}}}$$

The interaction between crystal grains takes place on grain boundaries, formed by the fusion of independently grown crystallites. The interaction on these boundaries explains the physical properties of polycrystalline material. There are several mechanisms of plastic deformation that have been shown for molecular crystals, but slip via formation and motion of dislocations is found to be the most abundant. Dislocations, in this case, are described as linear defects that create displacement in the long-range surrounding field. The possible mechanisms for the formation of molecular crystals’ physical properties are diverse and are extensively described in the review by Olson et al. [16].

To date, however, there has been no conclusive evidence that all molecular crystals follow the Hall–Petch relation. Also, the wax may include both substances that follow and do not follow the Hall-Petch equation, such as hydrocarbons [17]. Nevertheless, use of this relation might be helpful to forecast and control texture properties of oleogels after different technological treatment. The primary goal of the paper is to explore the quantitative relationship between the yield strength and crystal size distribution of oleogels based on beeswax and its hydrocarbons separately. We also aim to determine the thermodynamic parameters of the oleogels’ microstructure and their role in the formation the texture properties of oleogels.

## 2. Results and Discussion

### 2.1. Microstructure Analysis

**Figure 1:**
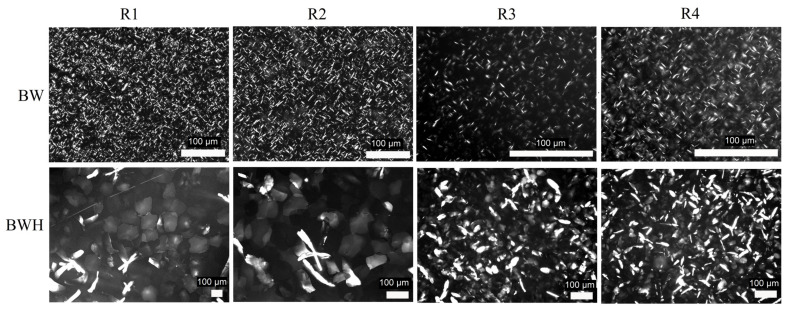
Representative microphotographs of beeswax and beeswax hydrocarbon-based oleogels prepared with different cooling regimes (Plan-Apochromat lens with 5× (BWH R1), 10× (BWH R2-R3), 20× (BW R1–R2), and 40× (BW R3–R4)).

Microscopy specimens were analyzed using polarized light microscopy. Representative microphotographs of the samples are shown in Figure 1. As can be seen from Figure 1, the shapes of the BW and BWH oleogels’ crystals have significant differences. Crystals of the BW oleogel at this magnification are seen as needles, however, it is a well-known misinterpretation of the microscopy data. It is shown that waxes form platelet-like crystals that are oriented perpendicularly between a glass slide and coverslip [18]. The top face is most likely to be {010} plane of the wax crystal [19]. This type of crystal orientation makes them visually brighter compared to the crystals with the {001} top face. The {001} oriented crystals are observed as hexagonal platelet in BWH oleogel at R1 and R2 regimes.

The changes in the shape of {001} oriented crystals should be mentioned. These crystals for BWH R1 and R2 oleogels have a more regular shape, which might be caused by differences in the crystal growth rate [20]. Also, it was observed that the amount of {010} oriented crystals was increased along with the cooling rate for BWH oleogel. In contrast, in BW oleogel the amount of {001} oriented crystals was increased along with the cooling rate.

**Figure 2:**
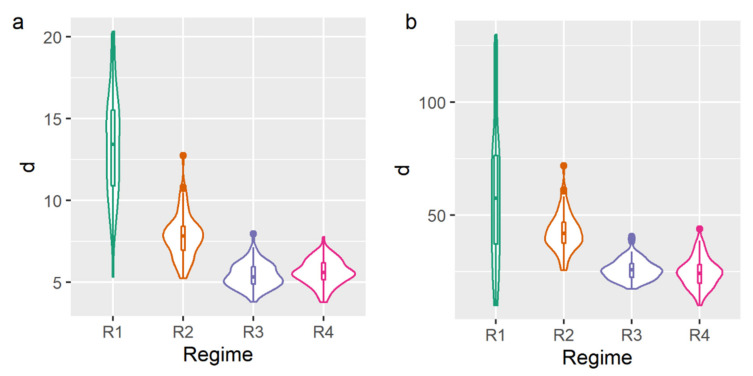
Crystal grain size distributions for beeswax-based (**a**) and beeswax hydrocarbons-based (**b**) oleogels prepared with different cooling regimes.

Figure 2a,b shows the crystal grain size distributions for the BW and BWH based oleogels accordingly. From this data, it can be seen that the crystal grain sizes vary within the range (from 1 μm to 1 mm) in which the Hall-Petch relation is known to be followed [15]. Based on this data, we have calculated the average grain size as the longest dimension. The results of the calculations for both samples are presented in Table 1.

As Table 1 shows, the average grain size decreases from R1 to R4 regimes. This phenomenon is expected and can be explained by the increase of the cooling rate that yields to increase in the nucleation rate and as a consequence of the decrease in the crystal size [21].

### 2.2. Microstructure Thermodynamic Parameters

Microstructure thermodynamic parameters were calculated according to the Section 2.2 and are shown in Table 2.

**Figure 3:**
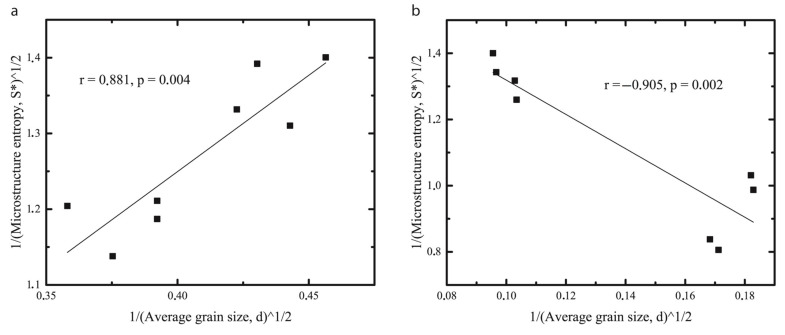
Relationship between microstructure entropy ($S_{m}^{*}$) and average grain size ($\bar{d}$) for beeswax (**a**) and beeswax hydrocarbons (**b**) based oleogels.

Contrary to the change of the average grain size, these parameters varied differently for the samples. $S_{m}^{*}$ of BW oleogels was decreased from R1 to R4 regimes, while $S_{m}^{*}$ of BWH oleogels increased. A positive linear relationship between 1/$\sqrt{S_{m}^{*}}$ and 1/$\sqrt{\bar{d}}$ observed for the BW oleogel (Figure 3a) is an expected outcome that might be observed for other systems [16]. Also, the same trend is expected for wax-based oleogels, since the smaller their crystals are in size, the more homogeneous they are distributed [8]. However, as shown in Figure 3b, this relationship is still linear but negative for the BWH oleogel. This finding is somewhat unexpected but seems to be consistent with a recent study, indicating that the mean crystal size is not linearly correlated with the variance of the crystal size (based on the measurement of the fractal dimension) over the wide range of cooling rates and crystal sizes [11]. Authors of this work have shown that at cooling rates higher than 0.35 °C/min, the variance of the crystal size distribution is increasing. This data can be related to our results, considering that the microstructure entropy used in our work is a function of a grain size distribution and is lower for distributions with a lower variation.

These findings may help to understand the mechanism underlying the change in the amount of {010} and {001} oriented crystals mentioned above. It was hypothesized by Blake and Marangoni [18] that {010} orientation is more preferable due to the lower Gibb’s free energy by minimization of the contact area between the hydrophobic wax and hydrophilic glass. In addition to this, grain boundary energy should also be considered as a parameter affecting crystal plane orientation [22].

Hence, two contact surfaces in a wax crystallization process are needed to be minimized: glass/“grain’s {001} plane” and “grain’s {010} plane”/“grain’s {010} plane” surfaces.

It can thus be suggested that if $\gamma_{gb}$ between grains’ {010} planes is lower than free energy between glass and grain’s {001} plane, the {001} orientation will be more preferable and vice versa.

Thus, considering that the free energy between the glass and the crystal grain is a constant for a given material, the probability of {010} and {001} orientations can be explained by increase or decrease of microstructure entropy accordingly as a function of a grain boundary energy.

### 2.3. Texture Properties of Oleogels

**Figure 4:**
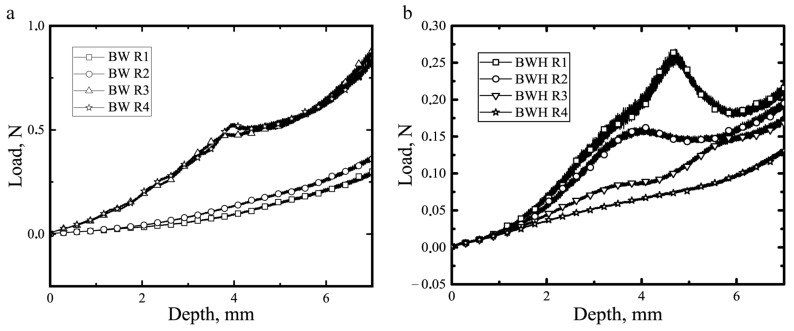
Characteristic load-displacement curves of beeswax (**a**) and beeswax hydrocarbons (**b**) based oleogels prepared with different cooling regimes.

The yield strength of oleogel is strongly affected by the cooling regime. The yield strength of oleogels crystallized under different cooling conditions was measured using a constant speed cone penetrometer test. An advantage of constant speed penetrometry compared to constant load penetrometry is a better control over the experimental parameters from the load-deformation response [23]. Figure 4 shows the experimental data from the cone penetration test.

As can be seen from Figure 4a, maximal load and yield strength during the test is significantly lower for R1 (0.27 N, 6.13 kPa) and R2 (0.23 N, 6.40 kPa) BW oleogels compared to R3 (0.75 N, 20.5 kPa) and R4 (0.78 N, 23.2 kPa) BW oleogels.

These results are consistent with data obtained by Hwang et al. [7] and confirm the positive association between the cooling rate and wax-based oleogel hardness. In contrast to this, BWH oleogels have shown the opposite relation. R1 (0.26 N, 10.2 kPa) and R2 (0.19 N, 8.35 kPa) BWH oleogels had a higher maximal load and higher yield strength than R3 (0.18 N, 3.43 kPa) and R4 (0.12 N, 2.49 kPa) BWH oleogels. The same dependency was observed by Venkatesan et al. [21]. Following their work, the yield stress of the paraffin gel also decreased with an increasing cooling rate. They have assumed that the formation of larger crystals results in a stronger gel, and therefore, a lower cooling rate results in the formation of stronger gels. This consideration explains the paraffin gel behavior but cannot be generalized to other wax-based oleogels.

These data were used to calculate microstructure thermodynamic parameters $\gamma_{s}$, $U_{m}$, $T_{m}$ (see Table 2) and also were used to evaluate whether the Hall-Petch ratio is observed for these systems.

### 2.4. Hall-Petch Relation for Oleogels

To describe the dependence of yield strength on the basic microstructural characteristics of average grain size and microstructure entropy, we have used the classical Hall-Petch relation (Equation (1)) and its modification (Equation (2)) accordingly.

**Figure 5:**
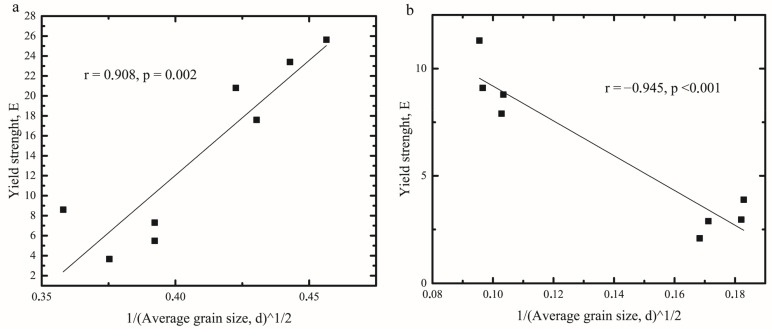
Relation between average grain size ($\bar{d}$) and yield strength (*E*) for beeswax (**a**) and beeswax hydrocarbons (**b**) oleogels.

Figure 5 shows plots calculated from the Hall-Petch relation for BW oleogels. The results both for the BW (Figure 5a) and BWH (Figure 5b) oleogels tend to fall along a line with high correlation coefficients (0.908 and −0.945 accordingly) and significance (*p* < 0.005).

The slope k in the Hall-Petch relation characterizes the influence of the grain size on the grain boundaries hardening and is interpreted as the measure of stress required for flow propagation across them [13,14]. Equations (3) and (4) are the corresponding linear relationship between 1/$\sqrt{\bar{d}}$ and yield strength for BW and BWH oleogels.

(3) $$E_{BW}=-80.1+\frac{17.8}{\sqrt{\bar{d}}}$$

(4) $$E_{BWH}=17.3-\frac{81.1}{\sqrt[{}]{\bar{d}}}$$

As can be seen, BW oleogel shows a positive correlation, and BWH oleogel a negative one, which corresponds to the direct and inverse Hall-Petch effects in these samples. BWH oleogel has higher absolute values of the parameter k, indicating higher dependence of yield strength from the average grain size. These results are consistent with the measurement of the stiffness change at different cooling rates by Hwang et al. for different waxes [7] and with the measurement of the Yield Stress change at different cooling rates by Venkatesan et al. [21] for hydrocarbons. These observations support the hypothesis of direct Hall-Petch effect in wax-based oleogels and inverse for hydrocarbons.

As evidenced by the data in Equation (3), the friction stress ($E_{0}$) in the absence of grain boundaries (when dislocations glide on the slip plane) is −80.1 kPa. Negative values of this coefficient are often interpreted as “unphysical” [22], but occur quite often, especially in polymorphic solid solutions with structures of needle-like (plate-like) type due to the heterogeneity of crystal orientations [23]. As previously shown [24], most combinations of beeswax fractions form solid solutions with the above-mentioned types of crystals. Thus, negative $E_{0}$ values are acceptable for the studied samples.

The inverse Hall-Petch effect observed for BWH oleogel (Figure 3b) is commonly observed in metals with nanoscale crystals and has been interpreted through various models: the dislocation-based model, which assumes that dislocation motion is the main factor of plastic flow [25]; diffusion-based models assume that different diffusion processes are responsible for this effect [26]; this effect can be explained by grain boundary shearing as the dominant cause [27]; it can also be explained by considering a grain boundary and a grain-interior as two separate phases in the system [28].

At present, there is insufficient information to definitively answer the question of what is the main cause of the inverse Hall-Petch effect in nanocrystalline metals. Moreover, there is not enough data for a definitive answer regarding hydrocarbon crystals, for which the applicability of the Hall-Petch effect is studied for the first time. However, taking into account that hydrocarbon crystals are formed by weak hydrophobic interactions, the approach outlined by Carlton and Ferreira [29] seems to be the most suitable for this purpose. This model assumes that atoms on a dislocation core are absorbed by the grain boundary with a certain probability, since the larger the grain size, the larger must be the number of the interacting atoms in the grain boundary. Thus, for larger grain sizes, the probability for dislocation absorption by the grain boundary is lowered, and the yield strength is higher.

**Figure 6:**
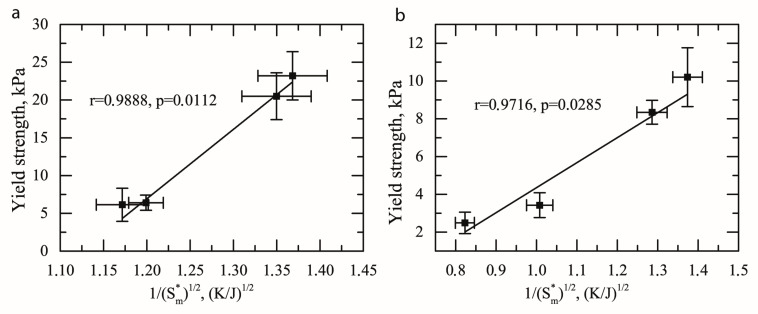
Relation between microstructure entropy ($S_{m}^{*}$) and yield strength (*E*) for beeswax (**a**) and beeswax hydrocarbons (**b**) oleogels.

By contrast, positive linear correlations can be seen on the plots calculated from the modified Hall-Petch relation for BW (Figure 6a) and BWH (Figure 6b) oleogels. High significance (*p* < 0.05) and correlation coefficients 0.989 and 0.972 for BW and BWH oleogels accordingly were also observed.

Equations (5) and (6) are the corresponding linear relationship between 1/$\sqrt{S_{m}^{*}}$ and yield strength for BW and BWH oleogels.

(5) $$E_{BW}=-87.3+\frac{79.7}{\sqrt{S_{m}^{*}}}$$

(6) $$E_{BWH}=-9.9+\frac{14.3}{\sqrt[{}]{S_{m}^{*}}}$$

Similarly, to the understanding of the slope k in the Hall-Petch relation, the slope in this modified equation might characterize the influence of the microstructure entropy on the grain boundaries hardening and is interpreted as the measure of stress, required for flow propagation across them [13,14]. BW oleogel, in this case, has higher values of the parameter k, indicating higher dependence of yield strength from the microstructure entropy.

The empirical grain boundary hardening coefficient k which represents the extent of the grain boundary obstacle effect against slip propagation is higher for BW oleogel (79.7 kPa × mm^1/2^), compared to BWH oleogel (14.3 kPa × mm^1/2^). Thus, the grain boundary obstacle effect is lower in BWH oleogel. This further supports the hypothesis on the absorption of dislocations by grain boundaries as a major factor causing the inverse Hall-Petch effect in BWH oleogel.

Another possible explanation for this is the relation of microstructure entropy with the grain boundary energy. Molecular crystals are characterized by anisotropy of intermolecular interactions and usually have stronger intramolecular and weaker intermolecular forces (in contrast to metals) [30]. For example, alkane crystals are formed by hydrophobic (non-bonded) intermolecular interactions and covalent (bonded) intramolecular interactions. Other (polar) components of BW, in addition to these forces, can interact through the electrostatic repulsion or attraction. Therefore, specific grain boundary free energy and bonding energy might play a higher role in the formation of the oleogel’s properties than grain size.

It can be thus suggested that microstructure entropy (in the case of wax based oleogels) is a more universal parameter, describing the correlation between microstructure and yield strength. A possible explanation for this might be that the microstructure entropy is not based on the absolute but relative (normalized) size values. This factor excludes the possible scaling effect on the results.

## 3. Conclusions

The relationship between yield strength and crystal size distribution of beeswax oleogels was investigated. In this study, we have used microstructure entropy ($S_{m}^{*}$) together with the average grain size ($\bar{d}$) as a basic microstructure parameters. This study has identified that microstructure entropy might be a useful parameter to explain the crystal morphology observed by polarized light microscopy. The research has also shown that the Hall-Petch equation can be used to describe the quantitative relationship between yield strength, inverse square-root of microstructure entropy, and average grain size. These experiments confirmed that hydrocarbons based oleogels follow the inverse Hall-Petch relation and indicated the direct Hall-Petch relation for beeswax-based oleogels. We have suggested that microstructure entropy might be a more universal microstructure parameter to characterize interactions in molecular crystals than average grain size. This is the first study to investigate the Hall-Petch effect in oleogels. The study, however, is limited by the range of crystal sizes and types of oleogelators used. The results of our study provide a theoretical basis for interpreting the microstructure and predicting the yield strength of beeswax oleogels. The model we applied evaluates the microstructure-dependent hardening coefficients and maximal hardening limits for different oleogels, which might be useful for fast and simple development of new oleogels with tailored properties.

## 4. Materials and Methods

### 4.1. Calculations

#### 4.1.1. Crystal Size Distribution

A total amount of 100 crystals for each sample was analyzed to represent the crystal distribution in the oleogel sample. Based on the obtained data we calculated the average grain size *d* using the formula shown in Equation (7)

(7) $$\bar{d}=\frac{d_{1}+d_{2}+\ldots +d_{n}+d_{N}}{N}$$

where $\bar{d}$ is an average grain size, $d_{i}$ is the *i*th grain size, and N is the total number of grains measured for the sample. Each grain size $d_{i}$ was normalized by average grain size to obtain dimensionless value ($d_{i}$/$\bar{d}$). Normalized grain sizes were then divided into bins (bin sizes 0.25, 0.4, 0.5, 0.6, and 0.75) to calculate the probability of finding grain in a particular bin ($p_{i}$). The probability was calculated as the ratio of the number of grains in the bin ($n_{i}$) to the total number of grains (N).

#### 4.1.2. Microstructure Thermodynamic Parameters

Crystal size distribution was used to calculate the microstructure entropy per grain by Equation (8), according to the approach described in [31].

(8) $$S_{m}^{*}=-\sum_{i}p_{i}\ln p_{i}$$

Entropy per grain $S_{m}^{*}$ for each sample was found as average over a range of all bin sizes to ensure that $S_{m}^{*}$ is invariant to bin size.

The microstructure entropy that is measured in this work is from the terms of the statistical thermodynamics. This understanding of entropy is described by Boltzmann and Gibbs [32], which defined entropy as a measure of the number of possible microscopic states of a system in thermodynamic equilibrium, which constitute the macroscopic state of the system. According to the third law of thermodynamics the entropy of a perfect crystal at absolute zero (0 K) is zero assumed. Therefore, this statistical entropy varies from zero to the theoretical maximum for each thermodynamic system. The lower the amount of the microstates of the system, the lower the entropy of the system.

Microstructure entropy according to [18] is in the direct correlation with grain boundary energy per unit volume by the usual thermodynamic relation (Equation (9))

(9) $$dS_{m}^{*}=\frac{dU_{m}}{T_{m}}$$

where $U_{m}$ (J) is a grain boundary energy per unit volume and $T_{m}$ (K) is the temperature of microstructure (averaged energy dissipation in slip avalanches [33]—the energy, that is required for transition of the crystal system from prior equilibrium state to a new local minimum under stress [34]). $U_{m}$ is calculated by Equation (10)

(10) $$U_{m}=\frac{\gamma_{gb}a}{\vartheta }$$

where $\gamma_{gb}$—is a grain boundary energy (J/m), a—is a grain area, and ϑ—is a grain volume. We have assumed that crystals have the shape of a parallelepiped, hence $a=2\left(x\cdot y+y\cdot z+x\cdot z\right)$ and ϑ=x·y·z, where *x*, *y*, and *z* are the mean dimensions of the crystals.

The grain boundary energy is a driving force for grain growth that can be calculated by Equation (11) [30]

(11) $$\gamma_{gb}=2\gamma_{s}-B$$

where *γ_s_* is surface energy and *B* is bonding energy.

The surface energy $\gamma_{s}$ is equals (*E*/8) × 10^–10^ m (E is the yield strength) as the elastic work done to create a free surface according to approximations made by Mullins [35]. Surface energy calculated by this method was used to estimate $\gamma_{gb}$ as $\gamma_{s}$/2 ≤ $\gamma_{gb}$ < $\gamma_{s}$.

### 4.2. Materials

Refined deodorized bleached sunflower oil (EFKO, Alexeevka, Russia) was purchased from a local store. Beeswax (BW) was provided by the local apiary (Nizhny Novgorod, Russia). Analytical grade acetone and hexane were purchased from Component Reaktiv (Moscow, Russia). DuraSil silica gel (pore diameter 120 Å, particle size 40–60 mm) for Dry Load Vessel was purchased from Elsico-HPLC (Moscow, Russia).

### 4.3. Hydrocarbons Separation

Beeswax hydrocarbons (BWH) were extracted by hexane according to the method described in [24] with minor modifications. We have used a Biotage Isolera Prime (Biotage, Uppsala, Sweden) preparative chromatography system equipped with a UV detector using Biotage SNAP 50g cartridge (dimensions: 39 × 81 mm, column volume 66 mL) with normal phase silica (45–50 mm particle size) (Biotage, Uppsala, Sweden). To perform the separation at room temperature, the dry loading method was used. To prepare the sample, 5 g of BW was dissolved in 20 mL of hexane-acetone (96:4 *w*/*w*). Fifteen grams of silica gel were added to the resulting solution. This mixture was dried on a rotary evaporator to a free-flowing powder state. The powder was then loaded into the column using the external dry load method using Dry Load Vessel (Biotage, Uppsala, Sweden). Before the separation cycle, the column was equilibrated with 2 column volumes of hexane. The eluent flow rate was 15 mL/min during the separation. The separation process was monitored at 205 nm against hexane as a blank. Eluate was collected and dried using a rotary evaporator to constant weight.

### 4.4. Oleogels Preparation

Samples of oleogels containing BW or BWH were produced by melting them in heated (90 °C) sunflower oil with mixing on a magnetic stirrer for 30 min. The concentration of gelling agents was 6% as an upper limit of critical gelling concentration for beeswax [36].

Melted at 90 °C, the oleogels were poured into the inversed cone sample holder (conical cup angle: 90°, depth: 20 mm), cooled to 25 °C, and thermostated for 24 h at this temperature before the analysis. To obtain samples with different crystal sizes, we used different cooling regimes. Each cooling regime was repeated twice, for both BW and BWH oleogels. Two slow cooling regimes with constant cooling rates equal to 0.5 °C/min (regime 1 or R1) and 1.0 °C/min (R2). Constant cooling rates for R1 and R2 were maintained in a climatic chamber Pol-Eko KK240 (Pol-Eko-Aparatura, Wodzisław Śląski, Poland). Also, we used two fast cooling regimes. For the first one, the samples were stored for 1 h at 4 °C (R3), and for the second, 1 h at −24 °C (R4). After this short-term cooling, samples were thermostated for 24 h at 25 °C in a climatic chamber.

In parallel, 2 mL aliquots of melted BW or BWH oleogels were mounted on the heated slide and covered with a coverslip. The specimens were cooled according to procedures R1–R4.

### 4.5. Texture Analysis

The samples in the reversed cone sample holder were then compressed by the cone probe (Conical Press opening angle: 90°, height: 20 mm), moving at a speed of 15 mm/s over a distance of 7 mm into the sample. The yield strength (E, kPa) and maximal load (F, N) were measured automatically by Trapezium X (Shimadzu, Tokyo, Japan) software [37].

### 4.6. Microscopical Analysis

The morphology of BW and BWH crystals in oleogels was studied using the Zeiss Axio Imager Z1 microscope in Polarized light mode (Carl Zeiss Microimaging GmbH, Jena, Germany). The images were taken using the Plan-Apochromat lens with 5×, 10×, 20×, and 40× magnifications according to the crystal size. Two replications of each sample were prepared for the crystal size analysis. At least two images of each replication were analyzed using ImageJ software.

### 4.7. Statistical Analysis

Statistical analysis was completed using Origin 2015. All measurement data were presented as means with standard deviation. Pearson coefficient was used to evaluate the correlations. Student’s *t*-test was used to compare the means between groups. Differences were considered significant at 5% (*p* < 0.05).

## Figures and Tables

**Table 1:** Crystal size parameters for beeswax and beeswax hydrocarbons based oleogels.

Sample	Cooling Regime	x, μm	y, μm	z, μm
BW	R1	7.46 ± 0.51	2.24 ± 0.15	2.10 ± 0.20 ^c^
	R2	6.51 ± 0.01	1.95 ± 0.01	2.40 ± 0.40 ^c^
	R3	5.25 ± 0.22 ^a^	1.57 ± 0.07 ^b^	0.80 ± 0.10 ^d^
	R4	5.22 ± 0.54 ^a^	1.56 ± 0.16 ^b^	1.00 ± 0.10 ^d^
BWH	R1	108.39 ± 1.74	32.52 ± 0.52	30.1 ± 0.20
	R2	94.07 ± 0.79	28.22 ± 0.24	12.3 ± 0.30
	R3	30.04 ± 0.18	9.01 ± 0.06	10.2 ± 0.10 ^e^
	R4	34.7 ± 0.81	10.41 ± 0.24	10.1 ± 0.10 ^e^

x, y, and z are dimensions of the crystals, shown as mean ± standard deviation. Samples without significant differences (*p* > 0.05) within dimension and gelator type are mentioned with the same uppercase letters.

**Table 2:** Microstructure thermodynamic parameters.

Sample	Cooling Regime	$S_{m}^{*},\;J/K$	γ_s_, J/m × 10^−8^	Um, J	Tm, K
BW	R1	0.73 ± 0.06 ^a^	7.66 ± 4.37 ^c^	0.048 ± 0.027 ^e^	0.066 ± 0.012 ^g^
	R2	0.70 ± 0.02 ^a^	7.99 ± 1.60 ^c^	0.053 ± 0.011 ^e^	0.076 ± 0.016 ^g^
	R3	0.55 ± 0.04 ^b^	25.6 ± 5.13 ^d^	0.188 ± 0.038 ^f^	0.342 ± 0.011 ^h^
	R4	0.54 ± 0.04 ^b^	29.0 ± 4.27 ^d^	0.213 ± 0.031 ^f^	0.394 ± 0.020 ^h^
BWH	R1	0.53 ± 0.03	12.8 ± 1.95 ^c^	0.050 ± 0.008 ^e^	0.094 ± 0.007
	R2	0.61 ± 0.04	10.4 ± 0.79 ^c^	0.041 ± 0.003 ^e^	0.067 ± 0.011
	R3	0.99 ± 0.06	4.28 ± 0.82 ^d^	0.019 ± 0.004 ^f^	0.019 ± 0.009
	R4	1.48 ± 0.08	3.11 ± 0.71 ^d^	0.013 ± 0.003 ^f^	0.008 ± 0.001

Samples without significant differences (*p* > 0.05) within parameter and gelator type are mentioned with the same uppercase letters.
